# CTX-M-14 β-lactamase-producing *Citrobacter freundii *isolated in Venezuela

**DOI:** 10.1186/1476-0711-10-22

**Published:** 2011-05-31

**Authors:** Beatriz Millán, Bárbara Ghiglione, Tulia Díaz, Gabriel Gutkind, María Araque

**Affiliations:** 1 Laboratorio de Microbiología Molecular, Facultad de Farmacia y Bioanálisis, Universidad de Los Andes, Mérida, Venezuela; 2 Cátedra de Microbiología, Facultad de Farmacia y Bioquímica, Universidad de Buenos Aires, Buenos Aires, Argentina; 3 Laboratorio de Síndromes Gastrointestinales y Urinarios, Facultad de Farmacia y Bioanálisis, Universidad de Los Andes, Mérida, Venezuela

**Keywords:** *Citrobacter freundii*, Extended-spectrum β-lactamases, CTX-M-14, plasmid, FrepB replicon

## Abstract

A clinical isolate of *C. freundii *with reduced susceptibility to extended-spectrum β-lactams from a woman with cystocele associated with recurrent urinary tract infection was analyzed. Susceptibility tests, double disk synergy tests (DDST) and enzymatic activity by the agar iodometric method suggested the presence of ESBLs. Conjugation experiments revealed the presence of a large conjugative plasmid (pLM07/20) with an exclusive FrepB replicon type (IncF/FIB). PCR analysis and sequencing confirmed the presence of the *bla*_CTX-M-14 _gene in the pLM07/20 from *C. freundii*.LM07/10. Although this is the first report of CTX-M-14 in Venezuela, we alert the medical community that future increase of these β-lactamases in our city could be due to dissemination of plasmids into bacterial populations.

## Background

Extended-spectrum β-lactamases (ESBLs) create significant therapeutic problems by inactivating almost all β-lactams except cephamycins and carbapenems. Following the detection of the first ESBL, TEM and SHV-derived ESBLs evolved and disseminated globally up to the late 1990s. Since then, a new epidemiological situation lead to a predominant increase of CTX-M derived enzymes [1]. Infections caused by ESBL-producing *Enterobacteriaceae *have mostly been described as hospital-acquired, associated with health care facilities [2], and, with increasing frequency, isolated from patients from both extended-care facilities and community-onset diseases [3,4]. *Citrobacter freundii *is an opportunistic pathogen that can cause diarrhea, septicemia, meningitis, and respiratory and urinary tract infection, especially in high-risk groups [5]. Multidrug resistance in *Citrobacter *spp. is a growing concern all over the world, and extended-spectrum cephalosporins are currently used to treat infections caused by these microorganisms. Numerous international molecular studies have also described the occurrence of various CTX-M types in species of *Citrobacter*, CTX-M-9 in the United Kingdom [6], CTX-M-30 in Canada [7], CTX-M-3 in Korea [8], CTX-M-2 in Japan [9], CTX-M-14 in China [10], and CTX-M-15 in India [11]. However, the distribution of CTX-M producers among community-onset pathogens from South America, especially in Venezuela, remains scarcely known. In this report, we describe a clinical isolate of *C. freundii *with reduced susceptibility to extended-spectrum cephalosporins that produces a CTX-M-14 type β-lactamase, from a woman with cystocele and urinary infection.

## Case presentation

In June 2010, a 47-year-old woman was assisted in private consulting with a history of grade II cystocele and recurrent urinary tract infection since December 2009. One cephalosporin-resistant strain of *Citrobacter freundii *LM07/10 was isolated from an urine sample. The strain was identified using conventional biochemical tests and confirmation was realized by API 20E system (bioMérieux, Marcy l'Etoile, France). The patient was treated according to the susceptibility test with i.v. meropenem. Also, a surgical treatment to repair the anterior vaginal wall prolapse was suggested.

Antimicrobial susceptibility testing was determined using the agar dilution method according to the recommendations of the Clinical and Laboratory Standards Institute (CLSI) [12]. The following antimicrobials (Valmorca, Mérida, Venezuela) were tested: cefoxitin, cefotaxime, ceftazidime, ceftazidime plus clavulanic acid, aztreonam, imipenem, meropenem, ciprofloxacin, gentamicin, amikacin and tobramycin. The ESBL phenotype was detected by the double disk (Oxoid, Basingstoke, Hampshire, England) synergy test (DDST) according to CLSI [12] guidelines. The quality control strains used were *Escherichia coli *ATCC 25922 (negative control for ESBL) and *Enterobacter cloacae *UBA-G CTX-M-9 (positive control for ESBL). β-lactamase presence in crude extracts obtained by sonication was confirmed by an agar iodometric method [13] with minor modifications [14] using cefotaxime as substrate.

To determine whether the resistance was transferable, conjugation experiments using mixed broth cultures were performed with *E. coli *J53 (azide^R^) as recipient. Transconjugants were selected on Levine agar (Britania, Buenos Aires, Argentina) plates containing 75 μg/ml of azide and 2 μg/ml of cefotaxime. The presence of *bla*_CTX-M _genes in the transconjugants was confirmed by DDST and PCR. Plasmid DNA from transconjugants was extracted by the rapid alkaline lyses method [15] and then analyzed by electrophoresis on a 0.8% agarose gel and visualized by staining with ethidium bromide under UV light. Identification of plasmids by PCR-based replicon typing was carried out as described by Carattoli et al. [16].

Detection of antimicrobial resistance *bla*_CTX-M _and *bla*_CTX-M-9 _genes were performed by PCR amplification using the following primers sets [17]: CTX-M-GF- 5'ATG TGC AGY ACC AGTA ARG TKA TGG C '3 and CTX-M-GR- 5' CCG CTG CCG GTY TTA TCV CCB AC '3 for *bla*_CTX-M_; CTX-M-9F- 5'AAC ACG GAT TGA CCG TCT TG '3 and CTX-M-9R- 5' TTA CAG CCC TTC GGC GAT '3 for *bla*_CTX-M-9_. The presence of the other β-lactamase genes was determined by previously described primers [11]. DNA templates were prepared by suspending one or two fresh colonies in 200 μl of sterile distilled water and heating at 95°C for 10 min. Amplification was performed in a final volume of 25 μl containing 1 μM of each primer, 0.2 mM of dNTPs, 1.5 mM MgCl_2 _and 1 unit of *Taq *polymerase. PCR products were visualized by electrophoresis on 1% agarose gels, stained with ethidium bromide (50 μg/ml) and photographed with UVP Biodoc-It System. Amplification products were purified (Sigma PCR Cleanup kit) and their nucleotide sequencing was performed with the ABI Prism 377 genetic analyzer (Applied Biosystems, CA, USA). Nucleotide and amino acid sequence alignments were analyzed using the Basic Local Alignment Search Tool (BLAST) suite of programs (http://www.ncbi.nlm.nih.gov/BLAST.cgi).

## Discussion

Recurrent urinary tract infection (UTI) remains an important public health problem in women of all ages [18]. We reported a case of a woman with grade II cystocele associated with recurrent UTI. This patient had more than three microbiologically documented episodes of symptomatic UTI during the last 6 months. Initially the patient was managed with empirical first line antibiotic therapy (amoxicillin or cephalexin) and later with extended-spectrum cephalosporins (ceftriaxone or cefotaxime). Recurrent infections are of particular concern because of the repeated morbidity and necessity for frequent administration of antibiotics with the possible adverse consequences of selection of antimicrobial-resistant strains.

A clinical isolate of *C. freundii *with reduced susceptibility to extended-spectrum β-lactam was collected from the patient urine sample. This isolate showed a multidrug resistance phenotype including ceftriaxone, cefotaxime, gentamicin, tobramycin and ciprofloxacin, but was completely susceptible to carbapenems. The DDST and enzymatic activity by the agar iodometric method suggested the presence of ESBL, which was confirmed by restored susceptibility to cefotaxime in the presence of clavulanic acid (Table 1). All resistance (or reduced susceptibility) markers were transferable by conjugation to recipient *E. coli *(J53 azide^R^), except ciprofloxacin. In this study, *C. freundii *harbored a plasmid of >150 kb with a FrepB replicon belonging to the IncF/FIB group. The identification of a family of related plasmids has important public health implications since it demonstrates that broad-host range plasmids carrying resistance to clinically relevant antibiotics can spread worldwide among bacteria responsible of both nosocomial and community-acquired infections [19].

**Table 1:** Antibiotic susceptibility (MIC) of ESBL-producing *Citrobacter freundii *LM07/10 and *Escherichia coli *J53-07/20 transconjugants.

Antibiotics tested	*C. freundii* LM07/10 MIC (μg/ml)	*E. coli *J53-07/20 transconjugants MIC (μg/ml)	*E. coli J53* MIC (μg/ml)	*E. coli* ATCC 25922 MIC (μg/ml)
**Cefoxitin**	8	8	4	4
**Cefotaxime**	> 16	8	0.064	0.125
Cefotaxime/clavulanic acid*	0.25	0.25	0.125	0.125
**Ceftazidime**	2	1	0.025	0.5
Ceftazidime/clavulanic acid*	0.25	0.25	0.25	0.125
**Aztreonam**	4	4	0.25	0.25
**Imipenem**	0.064	0.064	0.064	0.032
**Meropenem**	0.064	0.064	0.064	0.064
**Gentamicin**	> 64	32	0.5	0.5
**Amikacin**	8	4	0.5	1
**Tobramycin**	16	4	0.25	1
**Ciprofloxacin**	> 16	0.025	0.25	0.064
**Other test**				
DDST**	**+**	**+**	**-**	**-**

* Clavulanate was used at fixed concentration of 4 μg/ml.

** DDST: double disk synergy test.

Further DNA templates of *C. freundii *LM07/10, *E. coli *J53-07/20 transconjugants and plasmid (pLM07/20) were screened by PCR assays for *bla*_TEM_, *bla*_SHV _and *bla*_CTX-M _genes. Primers specific to the *bla*_CTX-M-9 _group gave positive gene amplification (850 bp fragment). Sequence analysis of the amplification product revealed the presence of *bla*_CTX-M-14_.

ESBLs-producing *Enterobacteriaceae *were previously identified in the small number of studies of venezuelan hospital-derivated isolates [20-23]. Previous reports of genotypes of ESBL genes from Venezuela were sporadic and involved SHV-5 and CTX-M-2, being particularly common in *Klebsiella pneumoniae *of nosocomial origin [20-22]. Also, the distribution of different genotypes of ESBLs in hospital or community in Venezuela is poorly understood. This is the first report of a CTX-M-14 β-lactamase producing *C. freundii *in Venezuela associated with a large conjugative plasmid with an exclusive FrepB replicon type (IncF/FIB) isolated from a community patient. But previous studies indicated that others replicons of different incompatibility groups (IncI or IncK) can also be involved in the dissemination of CTX-M-14 [24,25].

Recent studies suggest that infections due to ESBLs-producing organisms might be an emerging problem in outpatient settings in different countries [3,26]. Few data regarding the molecular epidemiology of CTX-M type ESBLs-producing *C. freundii *isolated from patient with community-onset infections are available [17,26]. According to the findings from this study, we may assume the hypothesis that microorganisms with antibiotic resistance mechanisms can be present in patients in the community and it is possible that healthy people could act as a reservoir of these ESBLs as a consequence of antibiotic use and abuse. Only through the recognition of factors associated with increasing resistance and the study of the molecular mechanisms involved, strategies can be traced and β-lactam resistance minimized.

In conclusion, this study supports the view that CTX-M-14 producing *C. freundii *is an emerging pathogen in community settings in Mérida, Venezuela, and that increased efforts in surveillance and study of risk factors associated with these bacteria are needed.

## Consent

Patient's written informed consent was obtained for publication of this case report. A copy of the written consent is available for review by the Editor-in-Chief of this journal.

## Competing interests

The authors declare that they have no competing interests.

## Authors' contributions

BM and BG carried out susceptibility test and molecular studies. TD performed isolation and microbiological identification of bacteria. GG and MA were involved in study conception and design, critical discussion, writing and review of the manuscript. All authors have read and approved the final manuscript.
